# Accuracy of Smartwatch Electrocardiographic Recording in the Acute Coronary Syndrome Setting: Rationale and Design of the ACS WATCH II Study

**DOI:** 10.3390/jcm13020389

**Published:** 2024-01-10

**Authors:** Mauro Buelga Suárez, Marina Pascual Izco, Jesús García Montalvo, Gonzalo Luis Alonso Salinas

**Affiliations:** 1 Cardiology Department, Hospital Universitario Ramón y Cajal, 28034 Madrid, Spain; maurobuelga@gmail.com (M.B.S.); jgarciamontalvo@gmail.com (J.G.M.); 2 Instituto Ramón y Cajal de Investigación Sanitaria (IRYCIS), 28034 Madrid, Spain; 3 Nursing and Physiotherapy Department, Universidad de Alcalá, 28805 Alcalá de Henares, Spain; 4 Cardiology Department, Clínica Universidad de Navarra (CUN), 31008 Pamplona, Spain; mpascualiz@unav.es; 5 Cardiology and Cardiovascular Surgery Department, Universidad de Navarra, 31006 Pamplona, Spain; 6 Cardiology Department, Hospital Universitario de Navarra (HUN-NOU), Calle de Irunlarrea, 3, 31008 Pamplona, Spain; 7 Navarrabiomed (Miguel Servet Foundation), IdiSNA, 31008 Pamplona, Spain; 8 Heath Sciences Department, Universidad Pública de Navarra (UPNA-NUP), 31006 Pamplona, Spain

**Keywords:** acute myocardial infarction, acute coronary syndrome, STEMI, NSTEMI, ECG tracing, ischemic heart disease, smartwatch

## Abstract

**Background**: Acute Coronary Syndrome (ACS), with or without ST-segment elevation, is a major contributor to global mortality and morbidity. Swift diagnosis and treatment are vital for mitigating cardiac damage and improving long-term outcomes. The 12-lead electrocardiogram (ECG) currently serves as the gold standard for diagnosis in ACS with ST-segment elevation and may support the diagnosis in ACS without ST-segment elevation. However, the growing prevalence of smartwatches enables the acquisition of electrocardiographic data without traditional ECG equipment. While smaller studies support smartwatch ECG use, larger-scale validation within ACS remains lacking. The ACS WATCH II study aims to validate smartwatch ECG recordings for ACS. **Methods**: The primary objective is to validate smartwatch-obtained electrocardiographic data in patients presenting with ACS. Two cohorts of 120 patients each, presenting ACS with and without ST-segment elevation, will be assessed. Smartwatches will capture recordings of leads I, III, and V2 alongside standard ECGs. These leads, chosen due to a 97% ACS diagnosis sensitivity in previous studies, will undergo blind evaluation by two experienced external assessors against conventional ECG. Additionally, a control sample of 60 healthy individuals will be included. **Conclusions**: ACS WATCH II pioneers large-scale prospective validation of smartwatch ECG recordings in ACS patients. Additionally, it indirectly validates a swift diagnostic approach using three leads (I, III, and V2). This could expedite time-critical ACS diagnoses and simplify access through smartwatch-based diagnosis.

## 1. Introduction

Acute Coronary Syndrome (ACS) stands as a prominent contributor to global morbidity and mortality, underscoring the paramount importance of early diagnosis and timely intervention to enhance patient outcomes. The electrocardiogram (ECG) serves as the pivotal diagnostic modality for ACS, enabling the detection of ST-segment elevation—an indicative marker of ST-segment elevation myocardial infarction (STE-ACS), necessitating urgent intervention. Furthermore, ECG can unveil anomalies, such as ST-segment depression or identification of negative T waves, associated with ACS without ST-segment elevation (NSTE-ACS), a potentially perilous condition. Nonetheless, conducting a conventional ECG promptly is not always feasible, especially in extra-hospital settings or resource-limited environments. In this context, smartwatches have emerged as a promising alternative for real-time electrocardiographic monitoring [1,2,3,4].

Numerous studies have appraised the precision of smartwatches in identifying ST- segment elevation among patients with STE-ACS. For instance, in a recent investigation, Spaccarotella et al. scrutinized a smartwatch’s capacity to detect ST-segment elevation in STEMI patients, yielding a sensitivity of 94.7% and specificity of 99.4% in comparison to conventional ECG [5]. However, additional studies are necessary to corroborate these outcomes and gauge the precision of smartwatches in patients with NSTE-ACS, a scenario in which there is no published evidence.

In this context, our research team recently executed a pilot study (ACS WATCH I) to assess the practicality of smartwatch recordings in select cases. The findings indicated that electrocardiographic recordings garnered through smartwatches hold potential as dependable aids for directing therapeutic interventions in Ischemic Heart Disease [6]. Nevertheless, a comprehensive validation study is imperative, given the limited scope of this pilot investigation.

Hence, this study aims to validate electrocardiographic recordings obtained from smartwatches in ACS patients, encompassing both STE-ACS and NSTE-ACS. A conventional 12-lead ECG will serve as the gold standard for comparison. This study, under no circumstances, aims to replace conventional ECG in diagnosis; it solely aims to be sensitive in expediting the diagnosis in specific scenarios and facilitating early access to specialized medical care. The anticipated result of this investigation is to enhance the management and care of ACS patients, offering a valuable alternative when access to a traditional ECG is restricted. Through achieving these objectives, this research endeavors to elevate the comprehensive care and outcomes of ACS patients, ultimately benefiting them in clinical practice.

## 2. Materials and Methods

### 2.1. Design

A prospective, multicenter, observational, and diagnostic comparison study will be conducted in two phases, one involving STE-ACS patients and another with NSTE-ACS patients.

Given that this is a validation study for an algorithm not previously validated with this device, a control sample will be included.

### 2.2. Setting

Recruitment will encompass individuals aged 18 and above with symptoms of ACS seeking medical attention at extra-hospital emergency services offered by SAMUR (Emergency and Rescue Municipal Assistance Service, Spanish initials) in the Community of Madrid, Ramón y Cajal University Hospital (Madrid, Spain), Arnau de Vilanova Hospital (Lleida, Spain), Hospital Universitari Dr. Josep Trueta (Girona, Spain), Clínica Universidad de Navarra (Madrid and Pamplona, Spain), and University Hospital of Navarra (Pamplona, Spain).

Control group individuals will be included after enrolling all patients with ACS, matching for age, body surface area, and gender.

### 2.3. Participants

Inclusion and exclusion criteria are shown in Table 1.

Patients meeting the inclusion criteria will be enrolled in the study after obtaining informed consent prior to their participation. If patients with STE-ACS require urgent cardiac revascularization, recruitment will occur in extra-hospital settings when symptoms manifest and ECG abnormalities are present. These individuals will be recruited from extra-hospital emergency services like SAMUR in Madrid or upon their arrival at tertiary hospitals before catheterization.

### 2.4. Procedures

#### 2.4.1. Conventional ECG Recording

All eligible patients must have a conventional ECG recorded within a maximum of 30 min prior to the electrocardiographic recording conducted using the smartwatch. The conventional ECG will be acquired following the standard practices or protocols applicable in each specific case.

Recruitment and the comparative conventional ECG recording in the control group will be conducted in the same manner.

#### 2.4.2. Smartwatch ECG Recording

Upon inclusion and after obtaining informed consent, all patients will have an electrocardiographic recording conducted by the smartwatch as follows:

Three recordings will be obtained: one for lead I, an additional one for lead III, and yet another for lead V2. This selection is based on the customary 30 s recording time for each lead. These specific leads were chosen after referring to previous studies [7], which demonstrated that electrocardiographic recordings from the lateral surface (lead I), the lower surface (lead III), and the front surface (lead V2) are generally effective for detecting ischemia in most instances.

The approach for recording each of these leads will be executed as follows:

**Lead I:** Recorded by positioning the smartwatch on the left wrist (back of the watch, positive electrode) and contacting the right index finger with the digital crown (negative electrode).

**Lead III:** Recorded by placing the smartwatch’s back (positive electrode) against the lower left abdomen, at the level of the iliac crest, and touching the digital crown with the left index finger (negative electrode).

**Lead V2:** Captured by placing the smartwatch’s back (positive electrode) on the left para-sternal line (4th intercostal space) and using the left index finger to touch the digital crown (negative electrode).

The procedure will be standardized and conducted by trained personnel to ensure consistent and accurate recordings. It will be the same for both the patient groups and the control group.

The smartwatches used for electrocardiographic recording will be Apple Watch Series 8 devices (Apple Inc., Cupertino, CA, USA).

#### 2.4.3. Smartwatch ECG Recording Analysis

Two independent researchers will compare the conventional ECGs and smartwatch ECGs. These researchers will not participate in patient recruitment or acquiring electrocardiographic recordings. The ECG assessment will be performed blindly, with the researchers lacking access to clinical data or other ECG results.

Both researchers will individually evaluate each ECG, and their conclusions will be juxtaposed. If discrepancies emerge between their interpretations, a third researcher will be consulted to resolve the disparities.

To enhance the reliability of interpretation, the criteria for ST-segment elevation and depression will be standardized as follows:

**ST-segment elevation**: ST-segment elevation will be considered if it is equal to or greater than 1 mm in leads I and III and equal to or greater than 2 mm in men and 1 mm in women, regardless of age, in lead V2.

**ST-segment depression**: ST-segment depression will be considered if it is equal to or greater than 0.5 mm.

#### 2.4.4. Storage of Recordings, ECGs, and Informed Consents

All participant records and data will be kept confidentially within a secure private cloud (Research electronic data capture—REDCap^®^, Vanderbilt University, Nashville, TN, USA), ensuring compliance with the Personal Data Protection Regulation (LOPD in Spanish initials). Patient data will be anonymized using specialized software to eliminate extraneous content like hidden text, metadata, comments, and attachments.

After transferring to the cloud, data access will be restricted solely to the principal investigator and study collaborators. Electrocardiograms and smartwatch records will be digitized and archived in PDF/JPG format. Each file will be identified by a unique participant identification number, recording date and time. The participants’ informed consent will also be digitized and stored in the same folder.

These records and data will be retained for a minimum of 10 years and then securely deleted in line with regulations governing personal data protection.

### 2.5. Outcomes

#### 2.5.1. Primary Outcome

The primary outcome of this study is the degree of concurrence in supporting the diagnosis of ACS, both with and without ST elevation, contingent upon the specific clinical scenario. This assessment will be conducted by comparing the result of the smartphone ECG rating and the actual 12-lead ECG, as evaluated by the two independent, blinded, and unbiased assessors. The concordance between observers will be quantified using the Kappa coefficient for inter-observer concordance.

#### 2.5.2. Covariables

Table 2 outlines the covariates that will be collected as part of this study.

### 2.6. Ethical Considerations

This study protocol has received approval from the Clinical Research Ethics Committee of the Ramón y Cajal University Hospital of Madrid (Code: 181/23).

### 2.7. Statistical Analysis

#### 2.7.1. Sample Size Calculation

Given the scarcity of comparable studies within the field, we have turned to previous investigations involving single-lead electrocardiographic recordings. For instance, the study conducted by Le KH et al. [7] in 2023, which employed a similar ECG interpretation approach utilizing three leads (lateral, inferior, and precordial), revealed an approximate sensitivity of 97%. Another relevant study by Spaccarotella et al. [5], conducted under analogous circumstances, reported sensitivities ranging from 93% to 99% and specificities between 85% and 99%. Drawing from these references, a cautious estimation was embraced, assuming a likely sensitivity of 95% and specificity of 85% for the smartwatch recording, thereby informing the rationale for determining the required sample size.

With sensitivity and specificity pegged at 100% for the conventional ECG (accounting for all cases diagnosed through conventional ECG), and a sensitivity of 95% coupled with a specificity of 85% for the smartwatch recording, alongside a significance level of 5% and power of 80%, an essential cohort of at least 104 patients within each group (those with and without ST-segment elevation) emerged as a prerequisite. This collates to a cumulative sample of 208 patients. Nevertheless, the sample size calculation is influenced by the limited evidence available. Thus, a margin of 15% was considered, increasing the total sample to 240 patients, with 120 in the NSTEMI group and 120 in the STEMI group.

In the case of the control group, we will include a ratio of 2:1 (case:control) to ensure comparability. A total of 60 healthy individuals will be included.

#### 2.7.2. Data Analysis

After data collection, a thorough statistical analysis will ensue to evaluate the effectiveness of smartwatch electrocardiographic recordings in detecting both STE-ACS and NSTE-ACS when contrasted with the conventional ECG. This analysis will extend beyond the computation of the Kappa coefficient for inter-observer concordance (primary outcome). It will encompass the calculation of sensitivity, specificity, positive predictive value, and negative predictive value for the smartwatch recording, with the conventional baseline ECG employed as the established reference standard. Comparisons will be conducted between the 12-lead conventional ECG and the simplified algorithm using the smartwatch, and between conventional ECG using only leads I, III, V2, and the smartwatch simplified algorithm.

To compare the diagnostic performance of both tests in identifying Acute Coronary Syndrome, Receiver Operating Characteristic (ROC) curves will be utilized. Furthermore, logistic regression analysis will be performed to identify variables associated with enhanced accuracy of the smartwatch recording. Statistical significance will be determined by a *p*-value of less than 0.05. All statistical analyses will be carried out using the Stata software (version 15, StataCorp LLC, College Station, TX, USA), facilitating precise and robust analysis.

## 3. Results

The results will be published as a peer-reviewed article.

## 4. Discussion

### 4.1. Anticipated Outcomes and Future Implications

The envisaged outcomes of this study encompass the validation of electrocardiographic recordings from smartwatches in patients afflicted by ACS, encompassing both cases with and without ST-segment elevation.

For patients displaying STE-ACS, it is foreseen that smartwatches will exhibit heightened sensitivity and specificity in the identification of this elevation. This advancement could substantially enhance the early recognition of individuals enduring acute myocardial infarction, consequently expediting their treatment. Conversely, among patients without ST-segment elevation, the integration of smartwatches is expected to expedite diagnoses in individuals who lack access to extra-hospital electrocardiography. This could engender the timely discernment of ECG irregularities, thereby enabling a swift and precise diagnosis, and guiding suitable medical intervention from the outset.

These findings bear profound practical implications. Initially, the deployment of smartwatches to detect ST-segment elevation in patients experiencing chest pain symptoms could function as a valuable instrument for the prompt identification of acute myocardial infarction, thereby augmenting treatment efficacy and prognoses. Secondly, the application of smartwatches to exclude ST-segment elevation in patients with chest pain symptoms devoid of this electrocardiographic indication might diminish the necessity for superfluous tests, consequently streamlining the diagnostic process.

Beyond the validation and assessment of smartwatch efficacy, the study also intends to indirectly authenticate a swift Acute Coronary Syndrome diagnosis via a 3-lead electrocardiographic recording: lateral, inferior, and anterior. This has the potential to revolutionize rapid ischemic heart disease diagnosis and amplify global diagnostic protocols.

Essentially, the validation of these 3-lead electrocardiographic recordings, obtained from smartwatches, for ACS has the capacity to notably enhance the benchmarks for managing and caring for patients dealing with this condition.

It is important to emphasize that this algorithm is solely designed for the early management of acute coronary syndrome in scenarios where a conventional 12-lead electrocardiogram is not available. This algorithm, under no circumstances, aims to replace conventional 12-lead ECG and only seeks to shorten response times and expedite referrals to tertiary centers. After the validation of this algorithm, it will be the responsibility of each center, community, or context to adapt this tool to their reality, thereby obtaining the maximum benefit possible.

### 4.2. Expected Limitations and Mitigation Strategies

Throughout the course of this study, we anticipate the presence of certain limitations that warrant acknowledgment. To counteract these challenges, we have devised the following measures:

**Ischemic changes in NSTEMI patients:** Unlike in STEMI, where clinical guidelines are clearer, ischemic electrical changes in NSTEMI patients tend to be more diffuse. Inclusion/exclusion criteria are designed to overlap with routine clinical practice, acknowledging that many patients with events may not exhibit electrical changes or may not meet these criteria. Therefore, the sensitivity calculated with our algorithm may not be applicable in this scenario, and the findings of this study will not be generalizable to the entire population with NSTEMI; it will only be applicable to patients exhibiting the ischemic changes mentioned in the inclusion criteria.

**ST-elevation criteria in V2:** The definition in the clinical guidelines for Acute Coronary Syndrome [8] is more complex than the criteria used in this study for event measurement. The clinical guidelines specify: ≥2.5 mm in men < 40 years, ≥2 mm in men ≥ 40 years, or ≥1.5 mm in women regardless of age. We simplify these figures by using a cutoff of ≥2 mm in men and ≥1 mm in women, regardless of age. This simplification is employed to achieve a highly sensitive algorithm, with less emphasis on specificity. However, by adding the control group, we will assess the validity of this approach, ensuring that sensitivity is maintained, and specificity is acceptable.

**Difficulty in Standardizing Data Collection**: Recognizing the potential for variability in data collection, we intend to preempt errors by implementing preemptive measures. To this end, prior to the study’s commencement, we will furnish participating personnel with instructional video tutorials, alongside an online tutorial conducted by the principal investigator. These resources will explicate the precise procedures for accurate data collection, ensuring a standardized approach.

**Time-Consuming Data Acquisition**: We acknowledge the potential for data acquisition to be time-intensive. To streamline this process, we will diligently train our research personnel in the proficient operation of the device. By doing so, we anticipate that the data collection time can be optimized. Each recording, encompassing all three derivations, is anticipated to take approximately 30 s, with an additional 60 s interval between each derivation for device calibration (it should be clarified that waiting for 60 s seems excessive, and after training, the time between taking two leads with the smartwatch should not exceed 5–10 s). As a result, the overall estimated time for complete data collection is around two and a half minutes for untrained personnel and less than 2 min for trained personnel.

Moreover, obtaining consent and a signature can further delay revascularization. Therefore, permission is obtained from the ethics committee to obtain verbal consent before revascularization, which will be formalized by filling out and signing the informed consent document after the procedure.

**Image Processing for Comparison**: Given the unique nature of the electrocardiographic trace, where each of the three derivations is stored as separate files, we recognize the need to accurately compare these files with the corresponding derivations of the standard 12-lead ECG. To address this challenge, we will institute a system wherein the PDF/JPG smartwatch ECG files and the conventional ECG will be stored within an individual folder for each patient. We will place special emphasis on meticulous classification during the training phase, ensuring that records are correctly categorized.

**Inability to generalize results to patients with pre-existing electrical changes:** Patients with left bundle branch block, pre-existing repolarization abnormalities, or stimulated by a pacemaker or resynchronization device are not included in this study. Therefore, the results are not generalizable to this population. In any case, conventional ECG also faces similar limitations in these patients, and prior probability, other complementary tests, and especially clinical presentation are crucial for making the diagnosis.

By proactively acknowledging these limitations and implementing tailored strategies, we are confident in our ability to navigate potential challenges and ensure the integrity of our study’s results.

## 5. Conclusions

With this study, the existing body of evidence concerning the utility of electrocardiographic recordings acquired via smartwatches in cases of Acute Coronary Syndrome (ACS) will be enhanced. Such enhancement holds the potential to catalyze a paradigmatic transformation and accelerate the provision of healthcare for this condition. This is especially pertinent in societal scenarios characterized by restricted availability of emergency medical services or inadequate medical infrastructure.

## Figures and Tables

**Table 1:** Inclusion and exclusion criteria. The table is divided according to the clinical scenario: STE-ACS (ST-Elevation Acute Coronary Syndrome) and NSTE-ACS (Non-ST-Elevation Acute Coronary Syndrome).

STE-ACS		NSTE-ACS	
Inclusion Criteria	Exclusion Criteria	Inclusion Criteria	Exclusion Criteria
Age ≥ 18 years. Symptoms of chest pain or equivalent, suggestive of ACS. ST-segment elevation of at least 1 mm in at least 2 contiguous leads in conventional ECG. Provide informed consent.	Left bundle branch block or previous ST-segment elevation/depression or negative T-waves on previous ECG. Pacemaker rhythm. Unable or unwilling to use the smartwatch. Do not provide informed consent. Hemodynamic instability or altered level of consciousness.	Age ≥ 18 years. Admitted for suspected ACS. ST-segment depression ≥ 0.5 mm or negative T-waves suggestive of ischemia, both in at least 2 contiguous leads in conventional ECG. Provide informed consent.	Left bundle branch block or previous ST-segment elevation/depression or negative T-waves on previous ECG. Pacemaker rhythm. Unable or unwilling to use the smartwatch. Do not provide informed consent. Hemodynamic instability or altered level of consciousness.

**Table 2:** Covariables. The table is divided according to the clinical scenario: STE-ACS (ST-Elevation Acute Coronary Syndrome) and NSTE-ACS (Non-ST-Elevation Acute Coronary Syndrome).

STE-ACS	NSTE-ACS
Age Sex History of hypertension History of diabetes mellitus History of previous coronary artery disease Present symptoms (chest pain, sweating, dyspnea, etc.) Smoking history Time from symptom onset to the smartwatch ECG recording Interpretation of the smartwatch ECG (presence of ST-segment elevation) Result of the conventional ECG (presence of ST-segment elevation) Time from symptom onset to the conventional ECG recording Angiographic findings in case of angioplasty or coronary revascularization surgery Troponin measurement	Age Sex History of hypertension History of diabetes mellitus History of previous coronary artery disease Present symptoms (chest pain, sweating, dyspnea, etc.) Smoking history Time from symptom onset to the smartwatch ECG recording Interpretation of the smartwatch ECG (presence of arrhythmias, changes in the T wave, etc.) Result of the conventional ECG Angiographic findings in case of angioplasty or coronary revascularization surgery Troponin measurement

## Data Availability

No new data were created or analyzed in this study. Data sharing is not applicable to this article.
